# Automated 3D Volumetry of the Pulmonary Arteries based on Magnetic Resonance Angiography Has Potential for Predicting Pulmonary Hypertension

**DOI:** 10.1371/journal.pone.0162516

**Published:** 2016-09-14

**Authors:** Fabian Rengier, Stefan Wörz, Claudius Melzig, Sebastian Ley, Christian Fink, Nicola Benjamin, Sasan Partovi, Hendrik von Tengg-Kobligk, Karl Rohr, Hans-Ulrich Kauczor, Ekkehard Grünig

**Affiliations:** 1 Department of Diagnostic and Interventional Radiology, University Hospital Heidelberg, Im Neuenheimer Feld 110, 69120 Heidelberg, Germany; 2 Translational Lung Research Center (TLRC), Member of the German Center for Lung Research (DZL), University of Heidelberg, Im Neuenheimer Feld 110, 69120 Heidelberg, Germany; 3 Department of Radiology, German Cancer Research Center (DKFZ), Im Neuenheimer Feld 280, 69120 Heidelberg, Germany; 4 Department of Bioinformatics and Functional Genomics, Biomedical Computer Vision Group, University of Heidelberg, BIOQUANT, IPMB, and German Cancer Research Center (DKFZ), Im Neuenheimer Feld 267, 69120 Heidelberg, Germany; 5 Radiology Isar Klinikum, Sonnenstrasse 22-24, 80331 Munich, Germany; 6 Clinical Radiology, Ludwig Maximilians University Hospital Munich, Marchioninistraße 15, 81377 Munich, Germany; 7 Institute of Clinical Radiology and Nuclear Medicine, University Medical Center Mannheim, Theodor-Kutzer-Ufer 1-3, 68167 Mannheim, Germany; 8 Department of Radiology, AKH Celle, Siemensplatz 4, 29223 Celle, Germany; 9 Department of Radiology, University Hospitals Case Medical Center, Case Western Reserve University, 11100 Euclid Ave, Cleveland, Ohio, United States of America; 10 Institute for Diagnostic, Interventional and Paediatric Radiology, University Hospital Bern, Inselspital, Freiburgstr. 10, 3010 Bern, Switzerland; 11 Centre for Pulmonary Hypertension, Thoraxklinik at the University Hospital Heidelberg, Röntgenstraße 1, 69126 Heidelberg, Germany; Banner Alzheimer's Institute, UNITED STATES

## Abstract

**Purpose:**

To demonstrate feasibility of automated 3D volumetry of central pulmonary arteries based on magnetic resonance angiography (MRA), to assess pulmonary artery volumes in patients with pulmonary hypertension compared to healthy controls, and to investigate the potential of the technique for predicting pulmonary hypertension.

**Methods:**

MRA of pulmonary arteries was acquired at 1.5T in 20 patients with pulmonary arterial hypertension and 21 healthy normotensive controls. 3D model-based image analysis software was used for automated segmentation of main, right and left pulmonary arteries (MPA, RPA and LPA). Volumes indexed to vessel length and mean, minimum and maximum diameters along the entire vessel course were assessed and corrected for body surface area (BSA). For comparison, diameters were also manually measured on axial reconstructions and double oblique multiplanar reformations. Analyses were performed by two cardiovascular radiologists, and by one radiologist again after 6 months.

**Results:**

Mean volumes of MPA, RPA and LPA for patients/controls were 5508 ± 1236/3438 ± 749, 3522 ± 934/1664 ± 468 and 3093 ± 692/1812 ± 474 μl/(cm length x m^2^ BSA) (all p<0.001). Mean, minimum and maximum diameters along the entire vessel course were also significantly increased in patients compared to controls (all p<0.001). Intra- and interobserver agreement were excellent for both volume and diameter measurements using 3D segmentation (intraclass correlation coefficients 0.971–0.999, p<0.001). Area under the curve for predicting pulmonary hypertension using volume was 0.998 (95% confidence interval 0.990–1.0, p<0.001), compared to 0.967 using manually measured MPA diameter (95% confidence interval 0.910–1.0, p<0.001).

**Conclusions:**

Automated MRA-based 3D volumetry of central pulmonary arteries is feasible and demonstrated significantly increased volumes and diameters in patients with pulmonary arterial hypertension compared to healthy controls. Pulmonary artery volume may serve as a superior predictor for pulmonary hypertension compared to manual measurements on axial images but verification in a larger study population is warranted.

## Introduction

Pulmonary hypertension (PH) is defined by an elevation of the mean pulmonary artery pressure ≥ 25 mmHg at rest on invasive right heart catheterization [1]. The condition carries a poor prognosis if it is not diagnosed and treated early [2,3]. However, prompt diagnosis remains challenging for various reasons [4]. The unspecific clinical presentation with symptoms such as dyspnea or fatigue does not directly suggest the diagnosis of PH [1]. Additionally, definite diagnosis requires right heart catheterization and thus an invasive procedure because non-invasive techniques have not proven sufficient accuracy yet [5–8].

Among other non-invasive methods, measurement of pulmonary artery diameters on axial computed tomography (CT) images has been suggested to assist diagnosis of PH [7,8]. Although pulmonary artery diameters measured on axial CT images are on average significantly increased in patients with PH, their sensitivity and specificity for predicting PH are only moderate [7–10]. An inherent limitation of measurements on axial CT images is that they are only a two-dimensional representation of three-dimensional geometries. Therefore, measurements derived from three-dimensional (3D) segmentation of pulmonary arteries might be more accurate for predicting PH.

3D segmentation of pulmonary arteries has been investigated in two previous studies based on CT data. The first study assessed the volume of intrapulmonary arteries, but not of the extrapulmonary arteries [11]. Although this study showed good correlation between intrapulmonary artery volume and pulmonary artery pressures, there was a significant overlap of intrapulmonary artery volumes in patients with and without PH thus hampering its usefulness for predicting PH [11]. The second study demonstrated the potential of 3D segmentation of the main, right and left pulmonary arteries by analyzing maximum diameters perpendicular to the respective vessel course, but did not investigate three-dimensional changes of pulmonary arteries [12]. To our knowledge, 3D analysis of the geometries of the main, right and left pulmonary arteries as well as 3D segmentation of pulmonary arteries based on magnetic resonance angiography (MRA) have not yet been published.

Purpose of this study was to demonstrate feasibility of 3D segmentation of central pulmonary arteries based on MRA, to assess 3D central pulmonary artery volumes in patients with PH compared to healthy controls, and to investigate the potential of the technique for predicting PH.

## Materials and Methods

### Subjects

The study was approved by the institutional review board of the University of Heidelberg and written informed consent was obtained prior to enrolment into the study. 20 consecutive patients (mean age 50 years, age range 19–72, 14 female, 6 male) with pulmonary arterial hypertension (WHO classification Group 1) confirmed by right heart catheterization (mean mPAP 53.7 ± 12.6 mmHg) undergoing MRA were prospectively included. Consecutive MRA acquisitions of 21 healthy controls without history of cardiovascular disease and with normal blood pressure (mean age 32 years, age range 21–51, 9 female, 12 male) served as reference. Mean body surface area (BSA) calculated according to the Du Bois and Du Bois formula was 1.79 ± 0.20 for patients and 1.84 ± 0.18 for healthy controls (p = n.s.) [13].

### Image Acquisition

**Fig 1:**
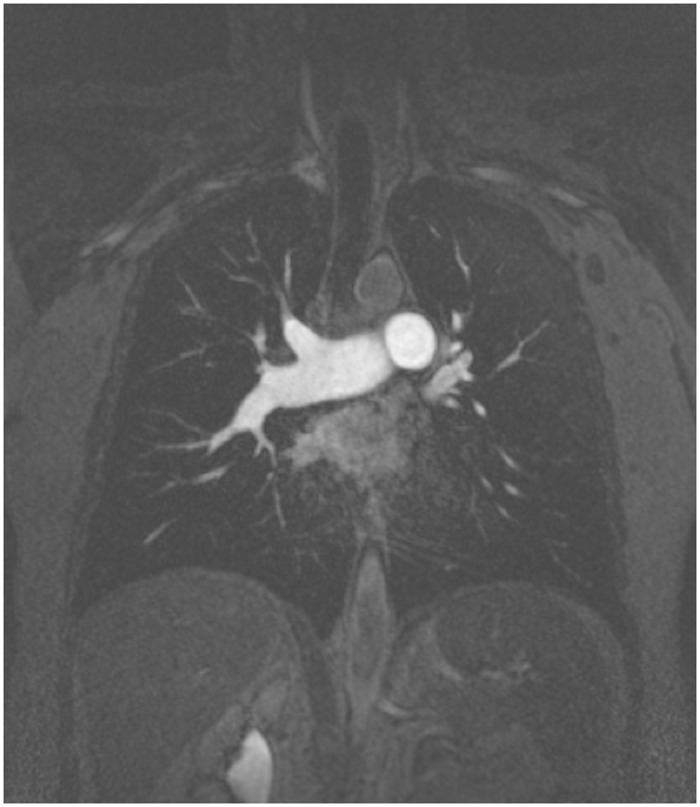
Example of image acquisition. Representative example of inspiratory breath-hold magnetic resonance angiography acquired in coronal orientation used for automated 3D segmentation of central pulmonary arteries.

A 1.5 T clinical MR scanner (Magnetom Avanto, Siemens, Erlangen, Germany) and a standard phased array body coil were used for image acquisition. For contrast-enhanced MRA, a three-dimensional T1-weigthed gradient echo spoiled FLASH sequence supplied by the manufacturer was applied in a coronal orientation (Fig 1). The following sequence parameters were used: slice thickness, 1.6 mm; in-plane resolution, 1.04 x 1.04 mm; repetition/echo time (TR/TE), 3.05/1.27 ms; flip angle 25°; parallel imaging factor 2; 120 slices per slab. Acquisition was performed in an inspiratory breath-hold. Gadolinium contrast agent was administered with a dose of 0.1 mmol/kg body weight via an ante-cubital vein at 2 ml/s followed by a saline flush of 20 ml at the same injection rate.

### Image Analysis

All image analyses were based on the unsubtracted MRA data. For assessment of interobserver agreement, all image analyses were performed by two cardiovascular radiologists. For assessment of intraobserver agreement, one of the two radiologists conducted image analysis for a second time after 6 months without reviewing the previous measurements. Each read was done in a different random order and blinded to the diagnosis.

Automated three-dimensional segmentation was performed using three-dimensional model-based image analysis software that was previously validated [14–16]. All information surrounding the software including the segmentation algorithm has been previously published [14–16]. In short, the software uses a three-dimensional analytic intensity model representing both shape and image intensities of vessels. This model is fitted to the image intensities within a volume of interest resulting in a three-dimensional model of the vessels of interest. The accuracy of the segmentation is improved by an automatic two-step refinement procedure.

**Fig 2:**
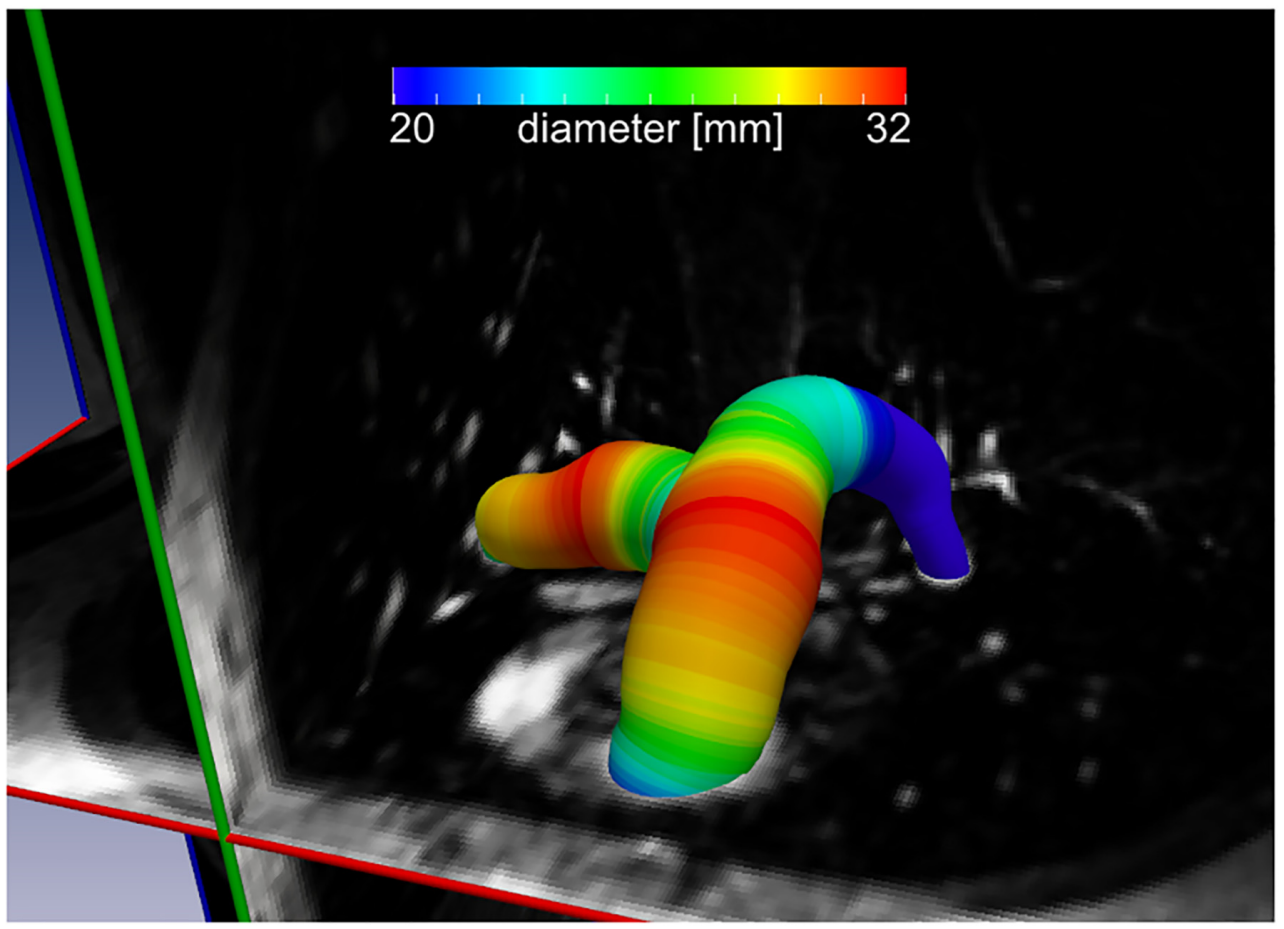
Example of 3D segmentation result. Representative automated 3D segmentation of central pulmonary arteries based on magnetic resonance angiography used for automated measurements. A color-coding is used to visualize pulmonary artery diameters of the 3D segmentation along the vessel course.

For this study, one seed point was placed in the center of the main, right and left pulmonary arteries, respectively (MPA, RPA and LPA). Starting at the seed point, the software automatically performed segmentation of the respective vessel both proximal and distal to the seed point and calculated centerlines along the geometric centroid of each segmented vessel (Fig 2).

The reader then visually verified the segmentation result and determined the level of the pulmonary valve, the MPA bifurcation and the take-off point of the upper lobe artery for both RPA and LPA. Pulmonary artery volumes were then computed by the software using the following standardized approach. MPA volume was defined as the volume between a level 5 mm distal to the level of the pulmonary valve and a level 5 mm proximal to the MPA bifurcation. RPA and LPA volumes were defined as the volumes between the start of the RPA/LPA at the MPA bifurcation and the take-off point of the right/left upper lobe artery, respectively. The respective vessel length was computed along the vessel centerline.

In addition to that, average vessel diameter perpendicular to the vessel centerline was automatically computed for every 1 mm along the centerline (Fig 2). Mean, minimum and maximum of the average diameter along the entire course of the respective artery were then also provided by the software. Three-dimensional segmentation and analysis took approximately 10 min per patient.

**Fig 3:**
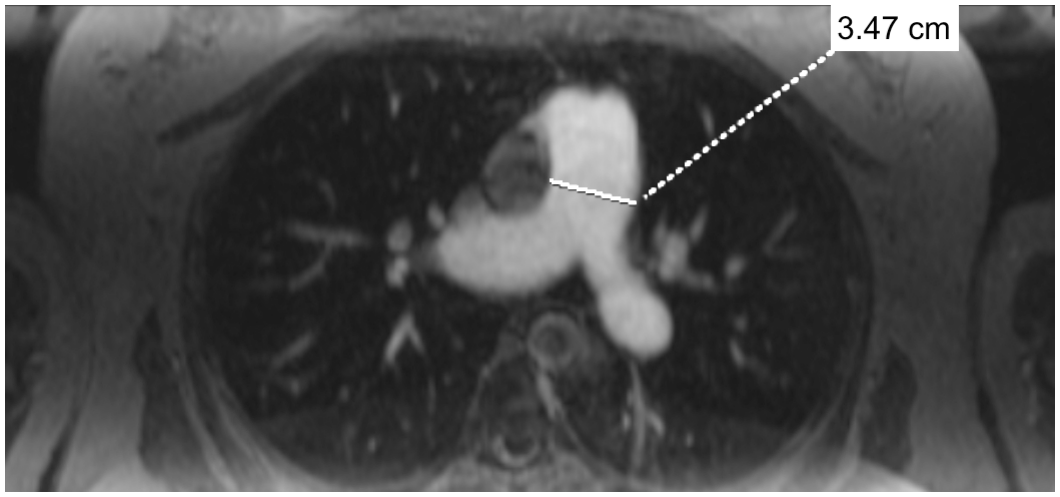
Example of manual measurement. Example of manual measurement of MPA diameter performed on axial reconstructions of the same magnetic resonance angiography data used for 3D segmentation. Manual measurements were performed for comparison with automated measurements.

For comparison purposes, the MPA, RPA and LPA diameters were manually measured on axial reconstructions at standardized locations. MPA diameter was measured at the level of the RPA as done in the Framingham Heart Study (Fig 3) [17]. RPA and LPA diameter were measured 1.5 cm distally to the MPA bifurcation. Axial reconstructions were obtained from the same MRA data sets used for 3D segmentation. These measurements were performed in a randomized order that was different to the randomized order for 3D segmentation by the respective reader again blinded to the diagnosis and also blinded to the results of the 3D segmentation.

Finally, the maximum MPA, RPA and LPA diameters were manually measured by the first reader using double oblique multiplanar reformations (MPR) using the same MRA data sets at the same standardized locations. Again, measurements were performed blinded to the diagnosis and also blinded to all other results and in a randomized order that was different to the randomized orders for 3D segmentation and axial reconstructions.

### Statistical Analysis

All volumes were indexed to the respective vessel length and corrected for BSA. All diameters were corrected for BSA. Shapiro-Wilk test was performed showing normal distribution of the analyzed data. Data is shown as mean ± standard deviation. The two-sided t-test for independent samples was applied to test for differences between PH patients and healthy controls. Inter- and intraobserver agreements were calculated as Shrout and Fleiss intraclass correlation coefficients. Overall agreement between measurements was assessed using Bland-Altman plots. The limits of agreement were calculated as mean ± 1.96 x standard deviation.

Area under the curve (AUC) using receiver-operating characteristic (ROC) analysis of average volume of RPA and LPA (VOL_AVG_) as well as their total volume (VOL_TOT_) was calculated to predict PH. For comparison, AUC was calculated for MPA diameter, average of RPA and LPA diameters (RPA/LPA diameter) and greatest pulmonary artery diameter measured on axial reconstructions and double oblique MPR.

A p-value of ≤ 0.05 was considered to represent statistical significance. All analyses were performed with SPSS Version 23.0 (SPSS Inc., Chicago, IL, USA).

## Results

Automated 3D segmentation was successful and deemed accurate in all cases apart from the MPA in one patient and the RPA in two healthy controls. In these cases, lower contrast in the pulmonary arteries compared to the ascending aorta secondary to suboptimal bolus timing was observed resulting in partial inclusion of the ascending aorta into the segmentation result. The affected segments were excluded from further analysis.

**Fig 4:**
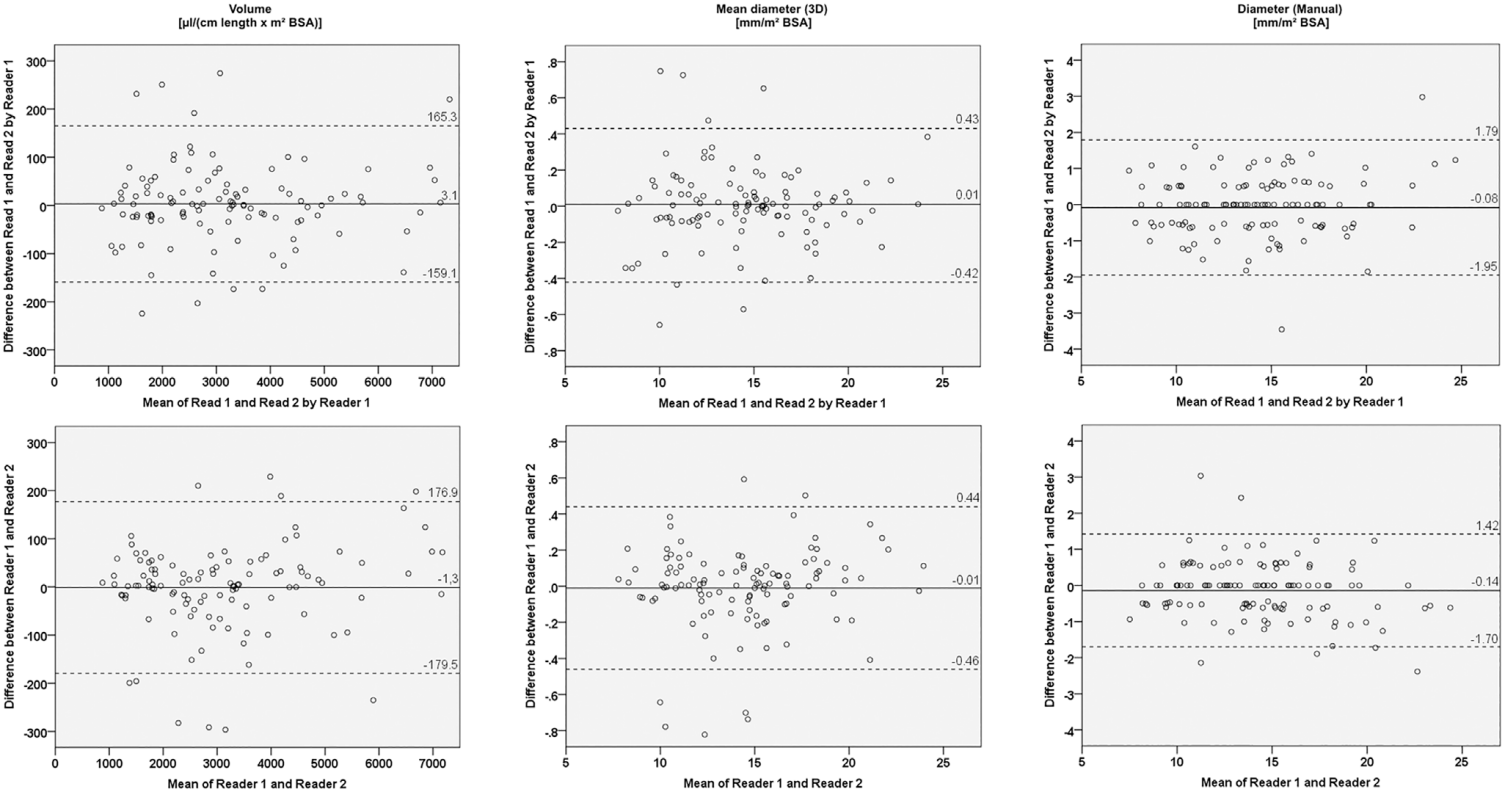
Bland-Altman plots for intra- and interobserver agreement. Bland-Altman plots show the differences between the two measurements performed by reader 1 (upper row) and the differences between measurements by the two readers (lower row) for automated 3D volume measurements (left), automated 3D mean diameter measurements (center) and manual diameter measurements (right) plotted against the means of the respective measurements. The straight line represents the mean difference, the dotted lines the limits of agreement. No systematic differences for repeated measurements could be observed. There is better agreement of automated 3D mean diameter measurements compared to manual diameter measurements.

Pulmonary artery volumes and diameters based on 3D segmentation were significantly increased in patients with PH compared to healthy subjects (Tables 1 and 2). Diameters manually measured on axial reconstructions and on double oblique MPR were also significantly increased in patients compared to healthy controls (Table 2). Measurements by 3D segmentation could be reproduced with excellent intra- and interobserver agreement (Tables 1 and 2). Bland-Altman plots did not show any systematic differences for repeated measurements (Fig 4). In comparison, intra- and interobserver agreement of manual axial MPA, RPA and LPA diameter measurements were slightly lower (Table 2), also visible in Bland-Altman plots (Fig 4).

**Table 1:** Pulmonary artery volumes indexed to vessel length and corrected for BSA [μl/(cm length x m^2^ BSA)].

Pulmonary artery	Patients	Healthy controls	P-Value	Intraobserver agreement	Interobserver agreement
Main	5508 ± 1236	3438 ± 749	<0.001	0.999*	0.998*
Right	3522 ± 934	1664 ± 468	<0.001	0.998*	0.998*
Left	3093 ± 692	1812 ± 474	<0.001	0.995*	0.995*

Data are means ± standard deviation calculated from means of measurements from all reads.

*P-values of intra- and interobserver agreement were all p<0.001.

**Table 2:** Pulmonary artery diameters corrected for BSA [mm/m^2^ BSA].

Pulmonary artery	Diameter	Patients	Healthy controls	P-Value	Intraobserver agreement	Interobserver agreement
Main	Mean_3D_	19.7 ± 2.2	15.4 ± 1.3	<0.001	0.998*	0.997*
	Min_3D_	18.1 ± 2.0	14.8 ± 1.2	<0.001	0.984*	0.977*
	Max_3D_	21.1 ± 2.6	16.4 ± 1.5	<0.001	0.993*	0.988*
	Manual_axial_	19.7 ± 2.6	14.4 ± 1.5	<0.001	0.962*	0.959*
	Manual_MPR_	20.1 ± 2.6	15.0 ± 1.5	<0.001	-	-
Right	Mean_3D_	15.7 ± 2.0	10.6 ± 1.6	<0.001	0.998*	0.998*
	Min_3D_	15.0 ± 1.9	10.0 ± 1.4	<0.001	0.997*	0.996*
	Max_3D_	16.9 ± 2.4	12.0 ± 2.0	<0.001	0.989*	0.994*
	Manual_axial_	15.6 ± 2.0	10.2 ± 1.5	<0.001	0.938*	0.965*
	Manual_MPR_	16.1 ±2.0	10.6 ± 1.5	<0.001	-	-
Left	Mean_3D_	14.8 ± 1.5	11.1 ± 1.5	<0.001	0.994*	0.994*
	Min_3D_	14.1 ± 1.6	10.0 ± 1.3	<0.001	0.986*	0.986*
	Max_3D_	15.6 ± 1.7	12.5 ± 1.7	<0.001	0.971*	0.987*
	Manual_axial_	15.0 ± 1.5	10.9 ± 1.6	<0.001	0.965*	0.965*
	Manual_MPR_	15.7 ± 1.6	11.3 ± 1.7	<0.001	-	-

Data are means ± standard deviation calculated from means of measurements from all reads. 3D indicates measurements by automated 3D segmentation, axial indicates manual measurements on axial reconstructions and MPR indicates manual measurements by double oblique MPR.

*P-values of intra- and interobserver agreement were all p<0.001.

AUC for predicting PH by VOL_AVG_ corrected for BSA was 0.998 (95% confidence interval 0.990–1.0, p<0.001) and by VOL_TOT_ corrected for BSA 0.997 (95% confidence interval 0.989–1.0, p<0.001). Sensitivity, specificity, positive predictive value and negative predictive value of VOL_AVG_ corrected for BSA were 100%, 95%, 95% and 100% using 2,500 μl/(cm length x m^2^ BSA) as sex-independent cut-off. For comparison, AUC for predicting PH by diameters manually measured on axial reconstructions was 0.967 for MPA diameter corrected for BSA (95% confidence interval 0.910–1.0, p<0.001), 0.990 for RPA/LPA diameter corrected for BSA (95% confidence interval 0.971–1.0, p<0.001) and 0.971 for greatest pulmonary artery diameter corrected for BSA (95% confidence interval 0.922–1.0, p<0.001). Sensitivity, specificity, positive predictive value and negative predictive value of RPA/LPA diameter corrected for BSA were 100%, 86%, 86% and 100% using 12.5 mm/m^2^ BSA as sex-independent cut-off. AUC for predicting PH by diameters manually measured on double oblique MPR was 0.953 for MPA diameter corrected for BSA (95% confidence interval 0.886–1.0, p<0.001), 0.986 for RPA/LPA diameter corrected for BSA (95% confidence interval 0.959–1.0, p<0.001).

## Discussion

MRA-based automated 3D segmentation of central pulmonary arteries is feasible and demonstrated significantly increased volumes and diameters of main, right and left pulmonary arteries in patients with pulmonary arterial hypertension compared to healthy controls. Measurements by 3D segmentation could be reproduced with excellent intra- and interobserver agreement. Furthermore, pulmonary artery volume showed higher sensitivity and specificity for predicting PH compared to pulmonary artery diameters manually measured on axial reconstructions.

These findings are well in accordance with previous studies investigating pulmonary artery diameters. Patients with PH have been shown to exhibit significantly increased central pulmonary artery diameters [18,19]. Besides, diameter measurements in healthy controls in our study are in the normal range established by previous studies [17].

Previous studies also demonstrated an only moderate predictive value of MPA diameter manually measured on axial images [7–10]. In our study, the predictive value of RPA/LPA diameter manually measured on axial reconstructions or double oblique MPR was higher compared to that of manual MPA diameters as well as greatest pulmonary artery diameter, but highest predictive value was achieved for pulmonary artery volume assessed by automated 3D volumetry. This may be explained by better depiction of the three-dimensional nature of geometric changes that pulmonary arteries undergo as a consequence of increased intraarterial pressures. Furthermore, quantifying geometric changes along the entire vessel course may be less prone to errors than measuring pulmonary artery diameters only at a single location. It has to be noted that measurements on axial imaging planes can be performed more easily in the clinical setting. Besides, inter- and intraobserver agreement and predictive value of axial measurements in this study might be considered sufficient for clinical routine. However, the additional effort of 3D volumetry may be justified by the improved predictive value. And although sensitivity and specificity for predicting PH will probably be lower both for manual axial measurements and automated 3D volumetry in a larger study population, it is likely that automated 3D volumetry will still be superior to manual measurements.

To our knowledge, this is the first study demonstrating the feasibility of MRA-based 3D segmentation of pulmonary arteries. Similar to CT, acquisition of MRA data can be performed noninvasively and in one breath-hold, but with the additional advantage of not requiring ionizing radiation. This might be particularly useful when performing repeated acquisitions for therapy monitoring. Further studies are needed to assess the value of the presented technique for therapy monitoring as well as predicting treatment response and overall survival, respectively.

It might be criticized that the healthy subject group included more males and had a lower mean age compared to the patient group. However, both males and younger subjects tend to have larger pulmonary arteries thus potentially reducing rather than increasing the differences between the PH patient and the healthy subject groups in the present study (4). Another limitation may be the relatively small number of subjects. However, the primary purpose of the present study was to demonstrate the feasibility and the potential of automated MRA-based 3D volumetry justifying a subsequent study in a larger patient population. Besides, inter-exam reproducibility was not investigated because we wanted to avoid repeated contrast medium administration within a short interval.

## Conclusions

Automated MRA-based 3D volumetry of central pulmonary arteries is feasible and demonstrated significantly increased volumes and diameters in patients with pulmonary arterial hypertension compared to healthy controls. Pulmonary artery volume may serve as a superior predictor for pulmonary hypertension compared to manual measurements on axial images but verification in a larger study population is warranted.

## Supporting Information

S1 Table Pulmonary artery volumes indexed to vessel length and corrected for BSA for all reads. Unit of all values is μl/(cm length x m^2^ BSA). Data are means ± standard deviation. Measurements are given for the two reads of reader 1 and the read of reader 2. Respective intra- and interobserver agreement are presented in the manuscript.
(DOCX)

S2 Table Pulmonary artery diameters corrected for BSA for all reads. Unit of all values is mm/m^2^ BSA. Data are means ± standard deviation. Measurements are given for the two reads of reader 1 and the read of reader 2. Respective intra- and interobserver agreement are presented in the manuscript.
(DOCX)

S3 Table Individual measurements of volume and axial diameter. Measurements are given for the two reads of reader 1 and the read of reader 2. Note that values given here are not corrected for BSA. Respective means as well as intra- and interobserver agreement are presented in the manuscript.
(DOCX)
