# Supplementary material for: Automated 3D Volumetry of the Pulmonary Arteries based on Magnetic Resonance Angiography Has Potential for Predicting Pulmonary Hypertension
Source: PLoS One. 2016 Sep 14;11(9):e0162516. doi: 10.1371/journal.pone.0162516 (PMC5023190; doi:10.1371/journal.pone.0162516)
Supplement: S1 Table — Unit of all values is μl/(cm length x m2 BSA). Data are means ± standard deviation. Measurements are given for the two reads of reader 1 and the read of reader 2. Respective intra- and interobserver agreement are presented in the manuscript. (DOCX) [file pone.0162516.s001.docx]

| **Pulmonary artery** | **Patients** | | | **Healthy controls** | | |
| --- | --- | --- | --- | --- | --- | --- |
|  | **Reader 1** | | **Reader 2** | **Reader 1** | | **Reader 2** |
|  | **Read 1** | **Read 2** |  | **Read 1** | **Read 2** |  |
| Main | 5517 ± 1268 | 5527 ± 1230 | 5479 ± 1211 | 3439 ± 761 | 3447 ± 750 | 3427 ± 743 |
| Right | 3507 ± 940 | 3517 ± 927 | 3541 ± 941 | 1675 ± 476 | 1653 ± 460 | 1664 ± 472 |
| Left | 3100 ± 716 | 3080 ± 694 | 3110 ± 685 | 1824 ± 524 | 1805 ± 447 | 1807 ± 462 |
