# Supplementary material for: Automated 3D Volumetry of the Pulmonary Arteries based on Magnetic Resonance Angiography Has Potential for Predicting Pulmonary Hypertension
Source: PLoS One. 2016 Sep 14;11(9):e0162516. doi: 10.1371/journal.pone.0162516 (PMC5023190; doi:10.1371/journal.pone.0162516)
Supplement: S2 Table — Unit of all values is mm/m2 BSA. Data are means ± standard deviation. Measurements are given for the two reads of reader 1 and the read of reader 2. Respective intra- and interobserver agreement are presented in the manuscript. (DOCX) [file pone.0162516.s002.docx]

| **Pulmonary artery** | **Diameter** | **Patients** | | | **Healthy controls** | | |
| --- | --- | --- | --- | --- | --- | --- | --- |
|  |  | **Reader 1** | | **Reader 2** | **Reader 1** | | **Reader 2** |
|  |  | **Read 1** | **Read 2** |  | **Read 1** | **Read 2** |  |
| Main | Mean_3D_ | 19.7 ± 2.3 | 19.7 ± 2.2 | 19.6 ± 2.2 | 15.4 ± 1.3 | 15.5 ± 1.3 | 15.4 ± 1.3 |
|  | Min_3D_ | 18.1 ± 2.0 | 18.1 ± 1.9 | 18.0 ± 2.0 | 14.8 ± 1.3 | 14.9 ± 1.2 | 14.7 ± 1.3 |
|  | Max_3D_ | 21.1 ± 2.6 | 21.2 ± 2.7 | 21.0 ± 2.6 | 16.4 ± 1.5 | 16.4 ± 1.7 | 16.4 ± 1.5 |
|  | Manual_axial_ | 19.6 ± 2.9 | 19.5 ± 2.4 | 20.1 ± 2.5 | 14.3 ± 1.5 | 14.6 ± 1.7 | 14.5 ± 1.6 |
| Right | Mean_3D_ | 15.7 ± 2.0 | 15.7 ± 2.0 | 15.8 ± 2.0 | 10.6 ± 1.6 | 10.6 ± 1.5 | 10.6 ± 1.6 |
|  | Min_3D_ | 14.9 ± 2.0 | 15.0 ± 2.0 | 15.1 ± 1.9 | 10.0 ± 1.5 | 10.0 ± 1.5 | 10.0 ± 1.5 |
|  | Max_3D_ | 16.9 ± 2.4 | 16.9 ± 2.4 | 16.9 ± 2.5 | 12.1 ± 2.1 | 12.0 ± 1.9 | 12.0 ± 2.0 |
|  | Manual_axial_ | 15.4 ± 2.0 | 15.7 ± 2.0 | 15.7 ± 2.0 | 10.1 ± 1.6 | 10.3 ± 1.7 | 10.2 ± 1.4 |
| Left | Mean_3D_ | 14.8 ± 1.6 | 14.7 ± 1.5 | 14.8 ± 1.5 | 11.1 ± 1.6 | 11.1 ± 1.4 | 11.1 ± 1.4 |
|  | Min_3D_ | 14.1 ± 1.7 | 14.0 ± 1.6 | 14.3 ± 1.7 | 10.0 ± 1.3 | 10.0 ± 1.3 | 10.1 ± 1.3 |
|  | Max_3D_ | 15.6 ± 1.7 | 15.6 ± 1.7 | 15.5 ± 1.7 | 12.6 ± 2.0 | 12.5 ± 1.6 | 12.2 ± 1.7 |
|  | Manual_axial_ | 15.2 ± 1.6 | 14.9 ± 1.5 | 15.0 ± 1.6 | 10.8 ± 1.8 | 10.9 ± 1.6 | 11.0 ± 1.6 |
