# Supplementary material for: Automated 3D Volumetry of the Pulmonary Arteries based on Magnetic Resonance Angiography Has Potential for Predicting Pulmonary Hypertension
Source: PLoS One. 2016 Sep 14;11(9):e0162516. doi: 10.1371/journal.pone.0162516 (PMC5023190; doi:10.1371/journal.pone.0162516)
Supplement: S3 Table — Measurements are given for the two reads of reader 1 and the read of reader 2. Note that values given here are not corrected for BSA. Respective means as well as intra- and interobserver agreement are presented in the manuscript. (DOCX) [file pone.0162516.s003.docx]

**A. Patients**

| **ID** | **BSA [m²]** | **Right pulmonary artery** | | | | | | **Left pulmonary artery** | | | | | |
| --- | --- | --- | --- | --- | --- | --- | --- | --- | --- | --- | --- | --- | --- |
|  |  | **Volume [µl/cm length]** | | | **Manual_axial_ [mm]** | | | **Volume [µl/cm length]** | | | **Manual_axial_ [mm]** | | |
|  |  | **Reader 1** | | **Reader 2** | **Reader 1** | | **Reader 2** | **Reader 1** | | **Reader 2** | **Reader 1** | | **Reader 2** |
|  |  | **Read 1** | **Read 2** |  | **Read 1** | **Read 2** |  | **Read 1** | **Read 2** |  | **Read 1** | **Read 2** |  |
| 1 | 1.74 | 5612 | 5914 | 5905 | 24 | 30 | 29 | 6228 | 6270 | 6224 | 28 | 28 | 28 |
| 2 | 1.58 | 6696 | 6640 | 6589 | 30 | 31 | 30 | 5323 | 5439 | 5625 | 27 | 26 | 29 |
| 3 | 1.51 | 3859 | 3855 | 3927 | 25 | 23 | 24 | 4064 | 4014 | 3989 | 25 | 24 | 24 |
| 4 | 1.68 | 9820 | 9694 | 10088 | 34 | 34 | 35 | 4535 | 4534 | 5024 | 28 | 26 | 25 |
| 5 | 2.13 | 9982 | 9776 | 9897 | 31 | 33 | 32 | 7042 | 7029 | 7026 | 31 | 31 | 31 |
| 6 | 1.83 | 5243 | 5501 | 5426 | 27 | 29 | 29 | 3848 | 4014 | 3933 | 21 | 21 | 21 |
| 7 | 1.90 | 5513 | 5697 | 6259 | 29 | 28 | 28 | 6392 | 6348 | 6355 | 28 | 27 | 29 |
| 8 | 1.70 | 5272 | 5141 | 5254 | 25 | 25 | 24 | 4503 | 4506 | 4732 | 25 | 23 | 24 |
| 9 | 1.96 | 8026 | 8076 | 8020 | 27 | 27 | 28 | 6296 | 6363 | 6259 | 28 | 28 | 28 |
| 10 | 2.27 | 10358 | 10338 | 10245 | 34 | 33 | 34 | 11212 | 11212 | 11194 | 37 | 38 | 36 |
| 11 | 1.96 | 5231 | 5306 | 5247 | 27 | 26 | 27 | 5033 | 4794 | 5091 | 28 | 26 | 26 |
| 12 | 1.59 | 4767 | 4659 | 4616 | 24 | 24 | 25 | 4846 | 4825 | 4852 | 25 | 25 | 24 |
| 13 | 1.79 | 5798 | 5812 | 5798 | 29 | 27 | 27 | 5114 | 5211 | 5094 | 28 | 27 | 27 |
| 14 | 1.62 | 4833 | 4661 | 4798 | 24 | 26 | 25 | 4465 | 4460 | 4490 | 25 | 23 | 24 |
| 15 | 1.92 | 5512 | 5413 | 5532 | 25 | 25 | 23 | 4543 | 4576 | 4609 | 24 | 24 | 24 |
| 16 | 1.56 | 2758 | 2809 | 2801 | 18 | 19 | 21 | 2767 | 2797 | 2801 | 21 | 22 | 22 |
| 17 | 1.89 | 6861 | 6873 | 6774 | 30 | 29 | 30 | 6421 | 6388 | 6378 | 28 | 28 | 27 |
| 18 | 1.78 | 7819 | 7945 | 7945 | 31 | 32 | 32 | 7237 | 7102 | 7143 | 31 | 31 | 31 |
| 19 | 1.75 | 5808 | 5806 | 5803 | 26 | 28 | 28 | 5757 | 5758 | 5769 | 25 | 26 | 27 |
| 20 | 1.62 | 6782 | 6985 | 6825 | 28 | 29 | 29 | 5758 | 5705 | 5771 | 28 | 27 | 28 |

**B. Healthy controls**

| **ID** | **BSA** | **Right pulmonary artery** | | | | | | **Left pulmonary artery** | | | | | |
| --- | --- | --- | --- | --- | --- | --- | --- | --- | --- | --- | --- | --- | --- |
|  |  | **Volume** | | | **Manual_axial_** | | | **Volume** | | | **Manual_axial_** | | |
|  |  | **Reader 1** | | **Reader 2** | **Reader 1** | | **Reader 2** | **Reader 1** | | **Reader 2** | **Reader 1** | | **Reader 2** |
|  |  | **Read 1** | **Read 2** |  | **Read 1** | **Read 2** |  | **Read 1** | **Read 2** |  | **Read 1** | **Read 2** |  |
| 1 | 1.60 | 2777 | 2714 | 2821 | 17 | 17 | 16 | 2759 | 2794 | 2775 | 16 | 18 | 16 |
| 2 | 1.88 | 3714 | 3675 | 3670 | 23 | 24 | 24 | 4048 | 4019 | 4550 | 24 | 23 | 23 |
| 3 | 1.83 | 4120 | 3947 | 4041 | 19 | 21 | 21 | 4144 | 3951 | 4130 | 21 | 21 | 20 |
| 4 | 1.82 |  |  |  | 18 | 19 | 20 | 3309 | 3215 | 3122 | 23 | 23 | 22 |
| 5 | 1.62 | 3556 | 3543 | 3560 | 21 | 21 | 20 | 5180 | 4737 | 4679 | 25 | 24 | 25 |
| 6 | 1.90 | 3114 | 2673 | 3045 | 20 | 19 | 19 | 3277 | 3552 | 3483 | 21 | 21 | 22 |
| 7 | 1.87 | 3652 | 3709 | 3593 | 19 | 19 | 19 | 4050 | 4041 | 4068 | 22 | 19 | 23 |
| 8 | 2.00 | 3776 | 3657 | 3665 | 21 | 20 | 20 | 4226 | 3726 | 3672 | 19 | 20 | 20 |
| 9 | 2.05 | 3667 | 3710 | 3636 | 20 | 19 | 20 | 3104 | 3565 | 3566 | 21 | 22 | 22 |
| 10 | 1.93 | 3012 | 3172 | 3127 | 21 | 19 | 20 | 2930 | 2968 | 2963 | 20 | 19 | 21 |
| 11 | 1.98 | 2463 | 2437 | 2472 | 15 | 16 | 17 | 3416 | 3366 | 3226 | 16 | 18 | 19 |
| 12 | 1.65 |  |  |  | 14 | 15 | 15 | 2069 | 2100 | 2428 | 15 | 15 | 16 |
| 13 | 2.02 | 2223 | 2216 | 2206 | 17 | 16 | 17 | 2439 | 2613 | 2648 | 17 | 18 | 18 |
| 14 | 1.79 | 2377 | 2304 | 2346 | 16 | 17 | 16 | 2967 | 2867 | 2768 | 18 | 19 | 18 |
| 15 | 1.98 | 5113 | 4897 | 5017 | 21 | 24 | 23 | 4721 | 4669 | 4651 | 23 | 25 | 25 |
| 16 | 2.13 | 2297 | 2506 | 2381 | 17 | 15 | 17 | 3816 | 3865 | 3749 | 21 | 20 | 21 |
| 17 | 2.07 | 4930 | 4979 | 5052 | 24 | 24 | 24 | 5560 | 5163 | 5131 | 28 | 27 | 27 |
| 18 | 1.61 | 2362 | 2358 | 2215 | 17 | 17 | 17 | 2320 | 2359 | 2188 | 17 | 18 | 17 |
| 19 | 1.54 | 2336 | 2307 | 2303 | 20 | 18 | 18 | 2330 | 2367 | 2259 | 16 | 17 | 16 |
| 20 | 1.84 | 1608 | 1619 | 1603 | 15 | 15 | 15 | 1867 | 2022 | 1979 | 17 | 15 | 16 |
| 21 | 1.65 | 2045 | 2002 | 2028 | 13 | 21 | 16 | 2347 | 2217 | 2214 | 16 | 18 | 17 |
